# Discovery of new butyrylcholinesterase inhibitors via structure-based virtual screening

**DOI:** 10.1080/14756366.2019.1644329

**Published:** 2019-07-26

**Authors:** Noor Atatreh, Sara Al Rawashdah, Shaikha S. Al Neyadi, Sawsan M. Abuhamdah, Mohammad A. Ghattas

**Affiliations:** a College of Pharmacy, Al Ain University of Science and Technology, Abu Dhabi, UAE;; b Department of Chemistry, College of Science, UAE University, Al-Ain, UAE;; c Department of Biopharmaceutics and Clinical Pharmacy, Faculty of Pharmacy, The University of Jordan, Amman, Jordan

**Keywords:** Butyrylcholinesterase inhibitors, Alzheimer’s disease, Ellman’s method, virtual screening, docking, pharmacophore

## Abstract

Butyrylcholinesterase (BChE) plays an important role in the progression of the Alzheimer’s disease. In this study, we used a structure-based virtual screening (VS) approach to discover new BChE inhibitors. A ligand database was filtered and docked to the BChE protein using Glide program. The outcome from VS was filtered and the top ranked hits were thoroughly examined for their fitting into the protein active site. Consequently, the best 38 hits were selected for *in vitro* testing using Ellman’s method, and six of which showed inhibition activity for BChE. Interestingly, the most potent hit (Compound 4) exhibited inhibitory activity against the BChE enzyme in the low micromolar level with an IC50 value of 8.3 µM. Hits obtained from this work can act as a starting point for future SAR studies to discover new BChE inhibitors as anti-Alzheimer agents.

## 1. Introduction

Alzheimer’s disease (AD) is one of the most prevalent neurodegenerative disorders that affect humans especially in older stage of their lives. It is estimated that the disease affected 50 million people worldwide according to World Alzheimer Report 2018 and the number is expected to grow to more than quadruple in the next 30 years^1^.

AD which is characterised by a gradual decline in cognitive function combined with behavioural and psychiatric symptoms^2^^,^^3^. Being multifactorial brain disorder, the exact pathophysiology of AD is not yet entirely known^4^. However, several pathogeneses of AD have been suggested: β-amyloid oligomerisation, τ-protein aggregation, cholinergic dysfunction, oxidative stress, and inflammation are implicated in the development of AD^5^^,^^6^. Most of the current treatments are based on the cholinergic dysfunction hypothesis, which states that degeneration of cholinergic neurons and a deficiency of the neurotransmitter acetylcholine (ACh) are the ultimate reasons for the loss of memory and decline of cognitive function^7–9^.

Cholinesterase is the enzyme which catalyses the hydrolysis of ACh and thus inhibition of cholinesterase can be an effective way for the treatment of AD^10^. It is well-known that two different cholinesterase (ChE) enzymes, acetylcholinesterase (AChE), and butyrylcholinesterase (BChE), are responsible for the hydrolysis of ACh within the brain^11^. However, AChE has stronger acetylcholine hydrolytic activity than BChE does under the same condition^12^, as former enzyme can cleave more than 10,000 ACh molecules per second^13^. AChE is substrate specific in nature and is found in high concentrations in the brain, while BChE is nonspecific and is distributed throughout the body^14^. In particular, it is associated with glial and endothelial cells in the brain^15^. In AD, the AChE level in the brain decreases progressively, but BChE level remains the same or increases up to 165% of the normal level^16^. This altered enzymes levels contribute to their different activity as the severity of AD advances^17^. ratio of BChE: AChE shifting from 0.6 to as high as 1.1 contributes to the formation of cholinergic deficits in these regions, leading to the behavioural, and cognitive dysfunction^18–21^.

Anti-cholinesterase drugs which are used in the treatment of AD are reversible protein inhibitors^22^. They used to postpone the onset of symptoms that are associated with patient lifestyle such as memory ability to think and speak, rationale judgment, and other thought processes^23^. Selective BChE inhibition is potentially advantageous for the treatment of AD. It circumvents the classical cholinergic toxicity that is a common side effect of AChE inhibition^24^.

Current Food and Drug Administration (FDA) approved cholinesterase inhibitors namely: donepezil^25^, rivastigmine,^26^ and galantamine^27^, help only in postponing the onset of the symptoms of AD and do not treat the underlying disease^28^.

In this study, we used a structure-based drug design approach to discover new BChE inhibitors which may serve as a valuable inspiration in the search for new treatment options of patients with advanced AD. First, an extensive literature survey was conducted to investigate the critical chemical features that contribute to the inhibition of BChE function and to better understand the binding fashion. Subsequently, we used structure-based virtual screening (VS) followed by Ellman’s method to determine the experimental activity of selected hits.

## 2. Methods

### 2.1. Molecular modeling

#### 2.1.1. Protein and ligand library preparation

The crystal structure of human BChE protein was obtained from the protein data bank (http://www.rcsb.org, PDB: 4TPK)^29^. All solvent molecules were eliminated then the MOE protein preparation module^30^ was used to check out the protein crystal structure for any missing atoms or residues and correct them accordingly^31^. The protein was then prepared by Protein Preparation Wizard of the Schrödinger modeling suite,^32^ that included adding hydrogen atoms to the protein structure and assigning partial charges to each atom.

Ligand database was downloaded from the NCI website (https://cactus.nci.nih.gov/download/nci/). The database was prepared by processing the molecules through LigPrep, the ligand preparation module in the Schrödinger software package^33^. The ligands were filtered based on drug-like rules; the Veber’s rules^34^ and Lipinski’s rule of five^35^. These filters included: molecular weight ≤ 500, hydrogen bond donor (HBD) ≤ 5, hydrogen bond acceptor (HBA) ≤ 10, logP ≤ 5, polar surface area (PSA) ≤ 140, and rotatable bonds ≤ 10.

#### 2.1.2. Virtual screening

The targeted BChE protein was subjected to a docking-based VS workflow using the docking module of the Schrödinger suite, Glide (Grid-based ligand docking with energetics)^33^. The prepared drug-like ligand library was docked into the active site of the protein. The Glide virtual screening workflow (VSW) is a three-step docking protocol involving three levels of increasing docking precisions: high-throughput virtual screening (HTVS); standard precision (SP); and extra-precision (XP). The 2000 ligands docked and ranked by GLIDE-XP were then clustered based on the MACCS algorithm^36^ and were then visually inspected. Finally, 38 ligands belonging to various structural scaffolds and showing convenient binding modes in the BChE active site were selected for biological assessment.

### 2.2. Biological assessment

#### 2.2.1. Quantification of BChE inhibitory activity in a spectrophotometric assay

Selected hits were obtained from NCI and they were provided as dry powders in variable quantities (5–10 mg). Compounds were initially dissolved in DMSO to give stock solutions of 100 µM. Subsequently, they were diluted to the required concentrations with Tris buffer pH 8.0 for the assay. Enzymatic inhibition assays were performed on BChE from equine serum (Sigma), according to the spectrophotometric Ellman's method^37^. The experiment was performed in 48-well plates in a final volume of 100 µL. Each well contained 0.22 U/mL eqBChE dissolved in Tris–HCl buffer, pH 8.0. They were preincubated for 20 min at different compound concentrations at 37 °C. Then 0.5 mM butyrylthiocholine iodide (Sigma) and 0.35 mM 5,5′-dithiobis -2-nitrobenzoico (DTNB; Sigma) were added. Colour development was measured spectrophotometrically at 412 nm using microplate reader (BioTek ELx800) at a rate of one measurement per minute over 15 min period. Positive (Eserine, sigma 100 uM) and negative (no inhibitors) controls were tested. All samples were assayed in at least duplicate measurements. In general, the amount of DMSO was kept below 1% in the assay. IC50 values were determined graphically from inhibition curves using Graph Pad prism version 6, Graph Pad Software, Inc..

### 2.3. NMR analysis of the top four hits

#### 2.3.1. Compound 4

**2-phenyl-9–(2-(pyrrolidin-1-yl)ethoxy)-4a,5-dihydro-2H-chromeno[4,3-c]pyridazin-3(4H)-one (602697):** ^1^H-NMR[DMSO-d_6_, 400 MHz]: (*δ, ppm*) 1.62–1.64 (m, 4H, 2CH_2_), 2.45 (m, 4H, 2CH_2_), 2.59 (m, 2H, CH_2_), 2.71 (m, 2H, CH_2_), 3.56–3.58 (m, 1H, CH), 3.88–4.45 (m, 4H, 2CH_2_), 6.89–6.91 (m, 1H, aromatic), 6.96–6.99 (m, 1H, aromatic), 7.27–7.32 (m, 2H, aromatic), 7.36 (brs, 1H, NH, exchanges with D_2_O), 7.41–7.45 (m, 2H, aromatic), 7.50–7.53 (m, 2H, aromatic).

#### 2.3.2. Compound 5

***(N^1^*-(7-chloro-3-methylquinolin-4-yl)-*N***^4^***,N^4^*-diethyl-2-phenylbutane-1,4-diamine (11052):** ^1^H-NMR[DMSO-d_6_, 400 MHz]: (*δ, ppm*) 0.91–1.02 (m, 6H, 2CH_3_), 1.84 (brs, 2H, CH_2_), 2.03 (brs, 2H, CH_2_), 2.17 (s, 3H, CH_3_), 2.34 (brs, 1H, CH), 2.75–2.90 (m, 4H, 2CH_2_), 3.62–3.76 (m, 2H, CH_2_), 5.42 (brs, 1H, NH, exchanges with D_2_O), 7.18–7.24 (m, 6H, aromatic), 7.77–7.78 (m, 1H, aromatic), 8.09–8.11 (d, 1H, aromatic, *J = 8.0 Hz*), 8.27–8.29 (m, 1H, aromatic).

#### 2.3.3. Compound 12

**2-[2-(benzylamino)ethylamino]-1,2-diphenylethanol (39813):** ^1^H-NMR[DMSO-d_6_, 400 MHz]: (*δ, ppm*) 3.08 (brs, 2H, CH_2_), 4.16 (brs, 2H, CH_2_), 4.57 (brs, 2H, CH_2_), 5.52 (brs, 1H, CH), 6.41 (brs, 1H, CH), 7.02–7.57 (m, 15H, aromatic), 9.53 (brs, 1H, NH, exchanges with D_2_O), 9.77 (brs, 2H, NH_2_^+^, exchanges with D_2_O), 10.44 (brs, 1H, OH, exchanges with D_2_O).

#### 2.3.4. Compound 26

**2–(2,3,4,9-tetrahydro-1H-carbazol-3-yl)ethanamine (135824):** ^1^H-NMR[DMSO-d_6_, 400 MHz]: (*δ, ppm*) 1.47 (m, 4H, 2CH_2_), 1.91–2.13 (m, 4H, 2CH_2_), 2.69 (m, 2H, CH_2_), 3.07 (brs, 1H, CH), 3.47 (brs, 3H, NH_3_^+^, exchanges with D_2_O), 6.92 (m, 2H, aromatic), 7.27 (m, 2H, aromatic), 10.61 (brs, 1H, NH, exchanges with D_2_O).

## 3. Results and discussion

Computer-aided molecular modeling protocol should involve sufficient study on the active site of the target enzyme before conducting the main VS^38^. Hence, the catalytic pocket of BChE protein was investigated and found to be consisting of a catalytic site at which the hydrolysis reaction takes place, its located at the bottom of a deep and narrow gorge, which is composed of conserved aromatic amino acids^39^. This most important site, also called the esteratic site, contains the three essential amino acids, Ser198, His438 and Glu325, which create the catalytic triad. They are involved in the transfer of the acetyl group from ACh to Ser198^39^. An essential role in the hydrolysis process is also played by amino acids in the BChE anionic site, which is responsible for binding the substrate quaternary ammonium group with cation–π interactions. Due to the interactions with the anionic site, the proper orientation of ACh in the gorge is provided.

One of the serine hydrolase features is stabilisation of the transition state by amino acids of the oxyanion hole through highly conserved N–H dipoles, derived from amino acids of the main chain: Gly116, Gly117, and Ala199^40^. During the enzymatic reaction, the transition complex is created and stabilised by those amino acids. The acyl pocket is responsible for substrate specificity. Comparison of human butyrylcholinesterase (HuBChE) and human acetylcholinesterase (HuAChE) shows differences in size, especially of the acyl binding pocket. The active gorge is larger in HuBChE than in HuAChE (500 Å^3^ versus 300 Å^3^). In HuBChE, the shape of the acyl pocket is determined by two residues: these are the aliphatic residues Leu286 and Val288, the presence of the smaller amino acids, valine and leucine, makes the hollow in the acyl pocket larger in BChE and enables larger molecules to get in^41^.

**Figure 1.**
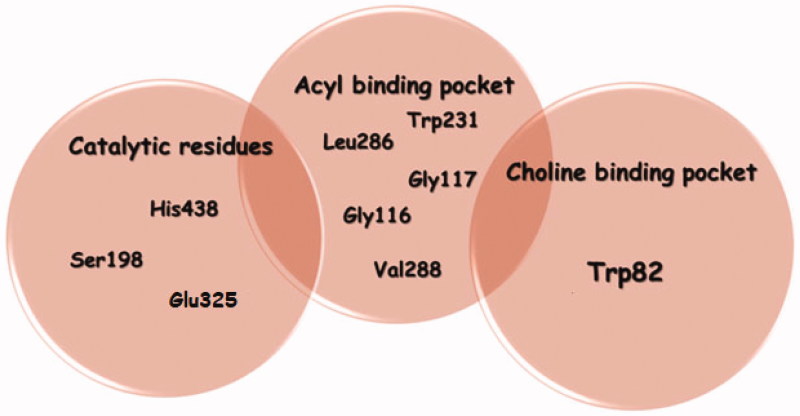
Main residues in the BChE protein binding site that are considered important in inhibiting the protein activity.

To sum up, the main residues in the active site were classified into three groups: catalytic residues (Ser198, His438, and Glu325), acyl binding pocket (Gly116, Gly117, Trp231, Leu286, and Val288), and choline binding pocket (Trp82) are shown below in Figure 1. After reviewing the literature, we can identify the following interactions in the active site as important for binding BChE inhibitors: interactions with the aromatic rings of Trp231, Trp82, and Phe329, to lesser extent H-bonding interaction with His438 and the tendency of the hit structure to enter the acyl pocket of BChE protein.

**Figure 2.**
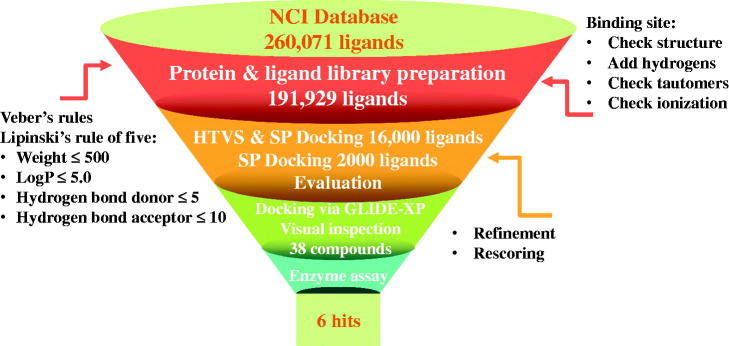
Schematic representation of the virtual screening protocol employed in the discovery of BChE inhibitors.

With regards to the BChE virtual screening, a schematic representation of the used protocol is shown in Figure 2. The VS started with filtering the NCI-ligand library according to drug-like characteristics The obtained drug-like ligand library where screened through three subsequent docking steps: HTVS, GLIDE-SP then GLIDE-XP so that docking precision increases gradually as ligands advance from one stage to another. The final outcome from VS process was filtered and preference was given to molecules containing features that are important for aforementioned interactions with active site residues.

To evaluate the inhibitory action of the top ranked hits, these compounds were ordered from NCI and were tested against the BChE protein using Ellman's method^37^. Eserine inhibitor was used as positive control. Table 1 shows the inhibition pattern of the top ranked compounds again the BChE enzyme. The IC_50_ values of these six hits were then identified as shown in Table 1. These active compounds exhibited variable inhibitory concentrations ranging from low to high micromolar range. Four out of these hits showed IC_50_ values below 100 µM (hit compounds 4, 5, 12, and 26). Interestingly, compound 4 exhibited the maximum inhibitor activity against BChE enzyme with an IC_50_ value of 8.3 µM.

**Table 1.** The IC_50_ values of the best compounds selected based on visual inspection inhibition activity of the tested top docked compounds using Ellman’s assay.

Hit compound	NCI Code	IC_50_ value (μM)	*R*^a^
3	NCS 23681	233.6 ± 1.22	0.9846
4	NCS 602697	8.3 ± 0.07	0.9848
5	NCS 11052	32.6 ± 0.11	0.9694
12	NCS 39813	22.2 ± 0.12	0.9701
25	NCS 162407	423.2 ± 1.19	0.9770
26	NCS 135824	39.7 ± 0.16	0.9912
Reference inhibitor	Eserine	0.04 ± 0.0001^b^	—

The regression coefficient of the dose–response curve as calculated by Graph pad prism 6.0. Inhibitory activity of the tested compounds was measured by half maximal inhibitory concentration (IC_50_).

a The regression coefficient of the dose–response curve as calculated by Graph pad prism 6.0.

b Reference inhibitor.

With regard to the *in silico* data, docking results for the six active hits are shown in Table 2. All active compounds exhibited low binding energies ranging from (−10.3 to −16.3 kcal/mol). These scores seem to be even more interesting if they got related to the size of the molecule since these hits scored ligand efficiency scores of around −0.5 kcal/mol. Ligand efficiency scores are calculated by dividing the docking score over the molecule weight of the compound and it indicates for suitability of the ligand to act as lead compound. Hence, these compounds look to have interesting binding energies with relatively small size when compared with the co-crystallised ligand.

**Table 2.** Docking scores and glide ligand efficiencies of the selected hits from the virtual screening and the co-crystallised ligand from the BChE protein that was used in the virtual screen.

Hit compound	NCI code	Chemical structure	Docking score (kcal/mol)	Glide ligand efficiency (kcal/mol)
3	NCS 23681		−16.28	−0.53
4	NCS 602697		−14.75	−0.51
5	NCS 11052		−14.70	−0.53
12	NCS 39813		−13.55	−0.52
25	NCS 162407		−12.86	−0.54
26	NCS 135824		−10.30	−0.64
Reference inhibitor	Co-crystallised ligand (4TPK)		−11.95	−0.36

**Figure 3.**
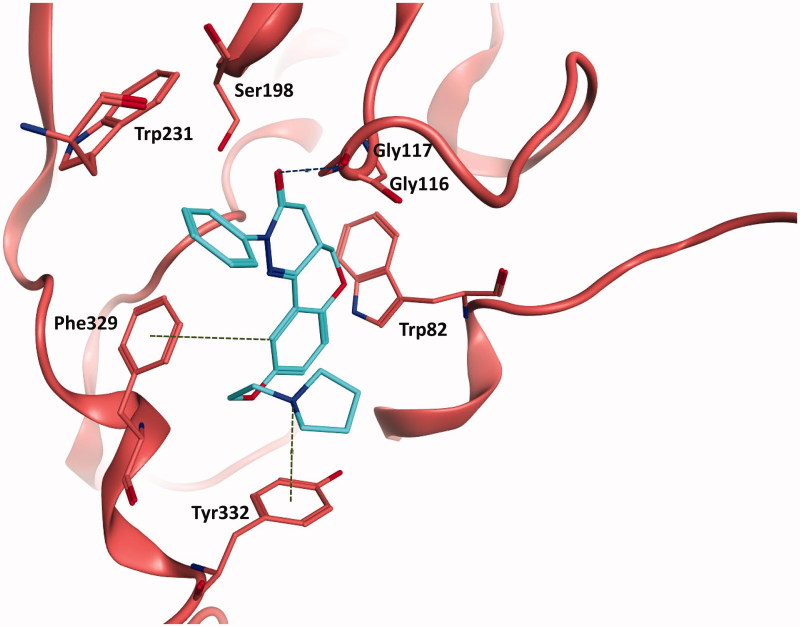
The docked binding mode of the most active hit, compound 4. The ligand is shown as cyan sticks and the protein is shown in as red cartoon and sticks. Hydrogen bonding is shown as blue dotted lines. Cation–π and hydrogen–π interactions are shown as green dotted lines.

**Figure 4.**
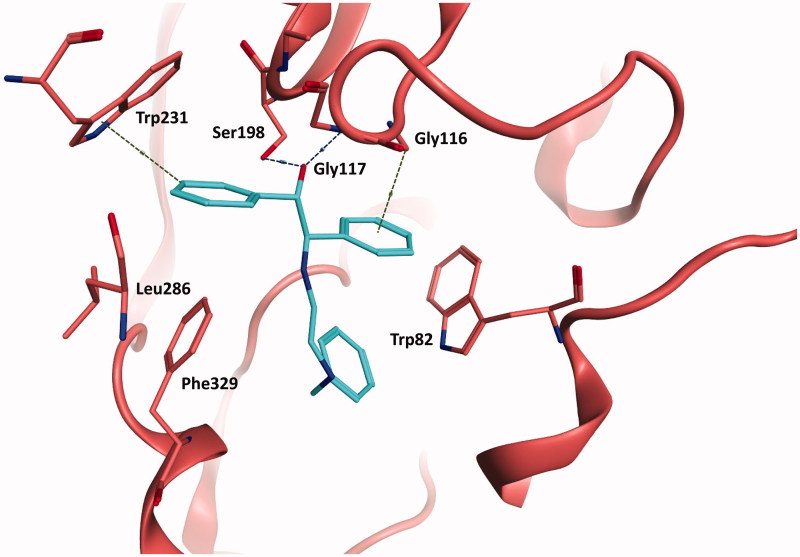
The docked binding mode of the most active hit, compound 12. The ligand is shown as cyan sticks and the protein is shown in as red cartoon and sticks. Hydrogen bonding is shown as blue dotted lines. Cation–π and hydrogen–π interactions are shown as green dotted lines.

Docked compounds showed convenient-binding modes in the BChE active site. In particular, to the best two hits (compounds **4** and **12**), the main interactions attributed to their activity relied on π–π and π–hydrogen interactions with Trp231, Trp82, and Phe329 along with hydrogen bonding with Gly117 and Ser198 (as shown in Figures 3 and 4). Additionally, the entry of the molecule within the acyl binding pocket of the active site along with the aforementioned bonding are believed to be substantially important for inhibitory activity.

^1^H-NMR spectroscopy was used to confirm the correct structure and the purity of the compounds. See spectra in supporting information. The structures of the compounds **4** were confirmed on the basis of ^1^H-NMR spectroscopy analysis. The ^1^H-NMR spectrum showed five characteristic signals resonates at *δ* = 1.62, 2.45, 2.59, 2.71, and 3.88 ppm attributed to the CH_2_ groups with an integration value of 16 protons. Another signal appeared at *δ* = 3.56 as broad band corresponding to CH proton. The eight aromatic protons resonated as a multiplet at *δ* = 6.89, 6.96, 7.27, 7.41, and 7.50 ppm. In addition, signal appeared at *δ* = 7.36 as broad band corresponding to NH-proton exchanged in D_2_O. The ^1^H-NMR spectrum of **5** showed a characteristic of two signals at *δ* = 0.91 and 2.17 ppm corresponding to the three methyl groups with an integration value of nine protons. The four signals attributed to the five CH_2_ groups resonates at *δ* = 1.84, 2.03, 2.75, and 3.62 ppm attributed to the CH_2_ proton with an integration value of 10 protons. While, NH proton resonated as a singlet at *δ* = 5.42 ppm and exchangeable with D_2_O. The aromatic protons resonated as a singlet at *δ* = 7.18, 7.77, 8.09, and 8.27 ppm with an integration value of nine protons. The ^1^H-NMR spectrum of **12** showed the correct chemical shifts of all protons of compound **12**. The ^1^H-NMR spectrum of **12** showed three multiplets resonated at δ = 3.08 ppm, 4.16 ppm and 4.57 ppm corresponding to six protons of CH_2_ group. While, two broad signals corresponding to CH groups appeared at δ = 5.52 and 6.41 ppm. The aromatic protons resonated as a singlet at *δ* = 7.02–7.57 ppm with an integration value of 15 protons. The ^1^H-NMR spectrum of **12** showed characteristic peaks at δ = 9.53 and 9.77 ppm due to –NH and NH_2_^+^ bands, respectively, and exchanged with D_2_O. while, the –OH proton resonated as a broad singlet at *δ* = 10.44 ppm. The structures of the compound **26** were clearly confirmed on the bases of ^1^H-NMR spectroscopy. The ^1^H-NMR spectrum of compound **26** showed three multiplet at *δ* = 1.47, 1.91 and 2.68 ppm, attributed to the corresponding five CH_2_ groups in the molecule. Another signal appeared at *δ* = 3.07 as multiplet corresponding to CH proton. A broad band at δ = 3.47 and 10.61 ppm corresponding to NH_3_^+^ and NH, respectively, both exchangeable with D_2_O. While, the aromatic protons resonated as a singlet at *δ* = 6.92 and 7.27 ppm with an integration value of 4 protons. Hence, all required integral values matches the number of protons without any other peak in the NMR spectra which confirm the purity of the compounds.

## Conclusion

Computer-aided drug design was employed to study and discover new BChE inhibitors that could be a starting point for a promising drug candidate in the treatment of AD. NCI database was filtered, treated, and subsequently screened against the BChE protein. Furthermore, the top docked compounds were tested against BChE activity which showed a direct correlation between the biochemical and drug design approaches by revealing four hits with IC_50_ values below 100 µM. The four hits’ identity was checked and their purity was confirmed. Compound 4 (IC_50_ value of 8.3 µM) and compound 5 (IC_50_ value of 32.6 µM) showed strong inhibitory activity that could become a starting point for future development and structure activity relationship studies for the discovery of new BChE inhibitors.

## Supplementary Material

Supplemental Material
