# Supplementary material for: Discovery of new butyrylcholinesterase inhibitors via structure-based virtual screening
Source: J Enzyme Inhib Med Chem. 2019 Jul 26;34(1):1373–9. doi: 10.1080/14756366.2019.1644329 (PMC6711031; doi:10.1080/14756366.2019.1644329)
Supplement: Supplemental Material [file IENZ_A_1644329_SM1356.docx]

**Supporting data for “Discovery of New Butyrylcholinesterase Inhibitors via Structure-Based Virtual Screening”**

Noor Atatreh^1^, Sara AlRawashdah^1^, Shaikha S. AlNeyadi^3^, Sawsan M. Abuhamdah^1,2*^, Mohammad A. Ghattas^1*^

^1^ College of Pharmacy, Al Ain University of Science and Technology, Abu Dhabi, UAE, P.O. Box 112612. Tel: +971 (0) 2 4444696. Fax: +971 (0) 2 4444304

^2^ Department of Biopharmaceutics and Clinical Pharmacy, Faculty of Pharmacy, The University of Jordan, Amman 11942, Jordan

3 Department of Chemistry, College of Science, UAE University Al-Ain, 15551 UAE

***List of figures:***

Figure 1**.** ^1^H-NMR spectrum for compound **4**

Figure 2**.** ^1^H-NMR spectrum for compound **5**

Figure 3**.** ^1^H-NMR spectrum for compound **12**

Figure 4**.** ^1^H-NMR spectrum for compound **26**

**
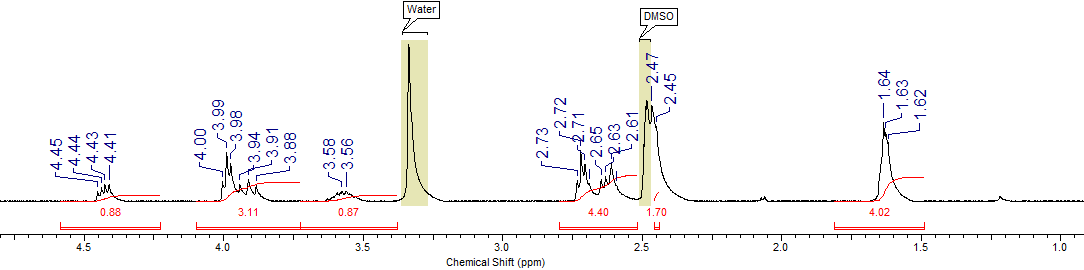
**
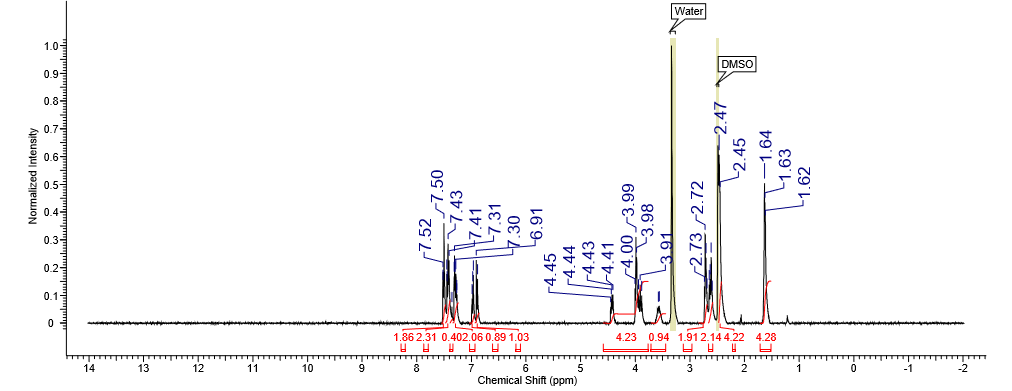


**Figure 1**. ^1^H NMR spectra of compound 4 (NCS **602697**) in DMSO-*d*_6_ at 298 K


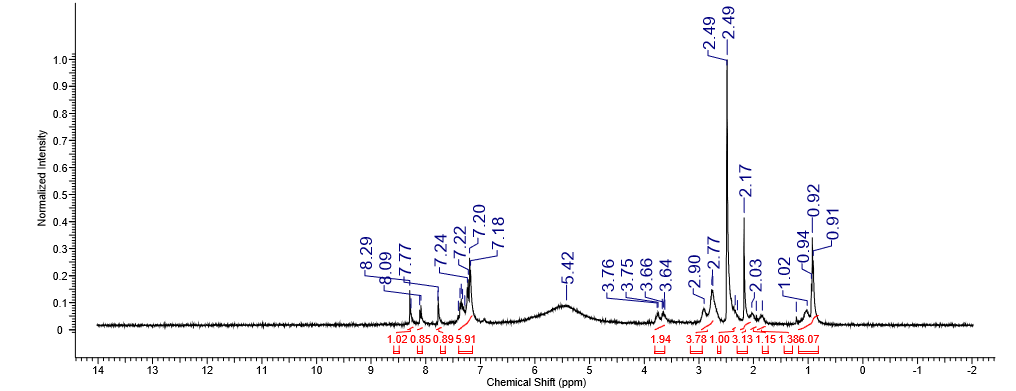


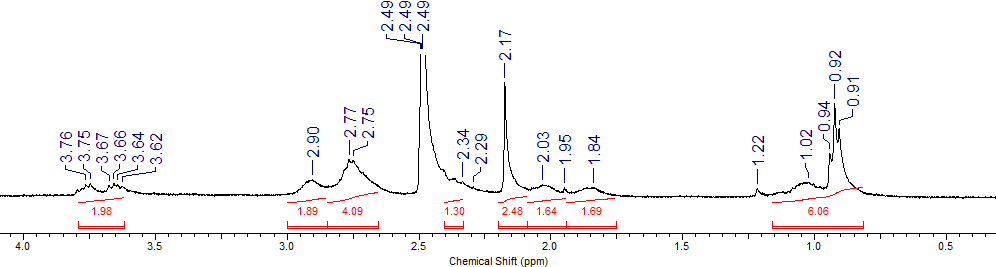


**Figure 2**. ^1^H NMR spectra of compound 5 (NCS 11052) in DMSO-*d*_6_ at 298


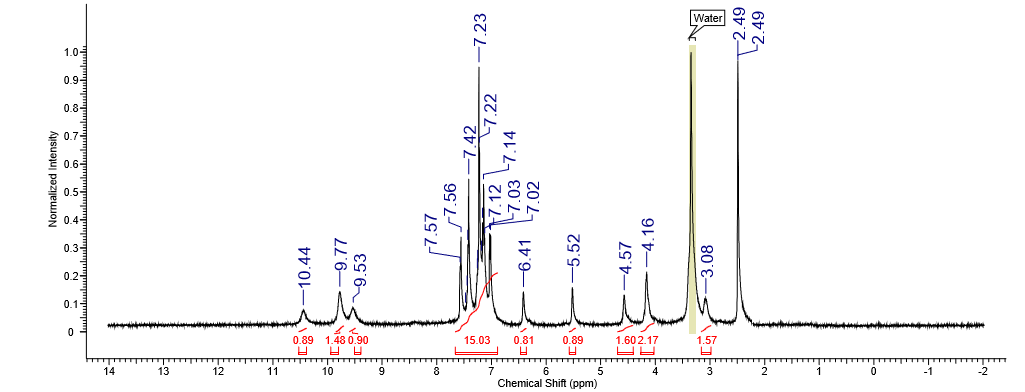


**Figure 1**. ^1^H NMR spectra of compound 5 (NCS 11052) in DMSO-*d*_6_ at 298 K

**Figure 3**. ^1^H NMR spectra of compound 12 (NCS 39813) in DMSO-*d*_6_ at 298 K


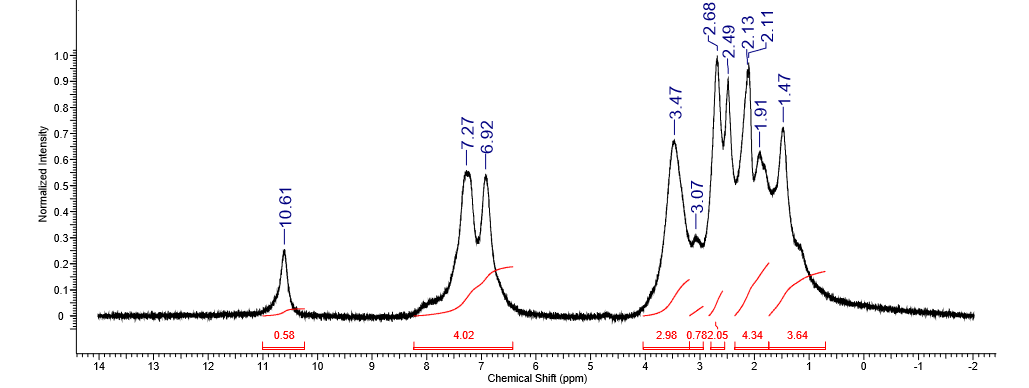


**Figure 4**. ^1^H NMR spectra of compound 26 (NCS **135824**) in DMSO-*d*_6_ at 298 K
